# The repetitive DNA sequence landscape and DNA methylation in chromosomes of an apomictic tropical forage grass, *Cenchrus ciliaris*

**DOI:** 10.3389/fpls.2022.952968

**Published:** 2022-09-15

**Authors:** Priyanka Rathore, Trude Schwarzacher, J. S. Heslop-Harrison, Vishnu Bhat, Paulina Tomaszewska

**Affiliations:** ^1^Department of Botany, Faculty of Science, University of Delhi, New Delhi, India; ^2^Department of Genetics and Genome Biology, University of Leicester, Leicester, United Kingdom; ^3^Key Laboratory of Plant Resources Conservation and Sustainable Utilization, Guangzhou, China; ^4^Key Laboratory of Applied Botany, South China Botanical Garden, Chinese Academy of Sciences, Guangzhou, China; ^5^Department of Genetics and Cell Physiology, Faculty of Biological Sciences, University of Wrocław, Wrocław, Poland

**Keywords:** repetitive DNA, methylation, retrotransposons, chromosomes, polyploidy, aneuploidy, breeding

## Abstract

*Cenchrus ciliaris* is an apomictic, allotetraploid pasture grass widely distributed in the tropical and subtropical regions of Africa and Asia. In this study, we aimed to investigate the genomic organization and characterize some of the repetitive DNA sequences in this species. Due to the apomictic propagation, various aneuploid genotypes are found, and here, we analyzed a 2*n* = 4x + 3 = 39 accession. The physical mapping of Ty1-*copia* and Ty3-*gypsy* retroelements through fluorescence *in situ* hybridization with a global assessment of 5-methylcytosine DNA methylation through immunostaining revealed the genome-wide distribution pattern of retroelements and their association with DNA methylation. Approximately one-third of Ty1-*copia* sites overlapped or spanned centromeric DAPI-positive heterochromatin, while the centromeric regions and arms of some chromosomes were labeled with Ty3-*gypsy*. Most of the retroelement sites overlapped with 5-methylcytosine signals, except for some Ty3-*gypsy* on the arms of chromosomes, which did not overlap with anti-5-mC signals. Universal retrotransposon probes did not distinguish genomes of *C. ciliaris* showing signals in pericentromeric regions of all 39 chromosomes, unlike highly abundant repetitive DNA motifs found in survey genome sequences of *C. ciliaris* using graph-based clustering. The probes developed from RepeatExplorer clusters gave strong *in situ* hybridization signals, mostly in pericentromeric regions of about half of the chromosomes, and we suggested that they differentiate the two ancestral genomes in the allotetraploid *C. ciliaris*, likely having different repeat sequence variants amplified before the genomes came together in the tetraploid.

## Introduction

*Cenchrus ciliaris* L. (buffelgrass, buffel-grass, or African foxtail grass; syn. *Pennisetum ciliare*) is an apomictic, allotetraploid perennial C_4_ grass native to tropical and subtropical arid regions of Africa and Western Asia. Predominantly distributed in warmer tropical regions, *C. ciliaris* grows in a range of harsh conditions (Kharrat-Souissi et al., 2012, 2013, 2014; Marshall et al., 2012). *Cenchrus* species are the predominant pasture grasses in India due to their high fodder value (Meena and Nagar, 2019). They are of great ecological importance due to persistence in native arid ecosystems, associated with tolerance to heavy grazing and drought, with deep root systems, and they are also used for erosion control (Miller et al., 2010). *C. ciliaris* is recognized as an invasive species in Australia (where it is a “declared plant”), South America, and the USA (particularly in Arizona and the Sonoran Desert) and has become a threat to natural ecosystems (Jackson, 2005; Miller et al., 2010) both by displacement of native plants and by intensifying wildfires. It is important to study the genetic characteristics and genomic organization of *C. ciliaris* to understand the association between biological, agricultural, and ecological characteristics.

*Cenchrus ciliaris*, with a base chromosome number of *x* = 9, includes tetra-, penta-, hexa-, and septa-ploid cytotypes (Visser et al., 1998; Kharrat-Souissi et al., 2012, 2013; Dhaliwal et al., 2018), and aneuploid accessions with 2*n* = 32, 40, 43, 44, and 48 have also been found (Burson et al., 2012; Carloni-Jarrys et al., 2018). Most accessions are apomictic, although sexual plants are also found occasionally in nature (Fisher et al., 1954; Marshall et al., 2012; Yadav et al., 2012), with a DNA-based SCAR marker reported by Dwivedi et al. (2007). Apomictic seed production preserves superior genotypes, including heterozygotes and polyploids (Spillane et al., 2004; Bhat et al., 2005; Abdi et al., 2016), and can discover new polyploid lineages. The characteristic is well-represented in polyploid grasses, including members of Paniceae grass genera such as *Panicum, Pennisetum, Cenchrus*, and *Paspalum* (Worthington et al., 2016; Higgins et al., 2021; Tomaszewska et al., 2021b). In *Urochloa* (*Brachiaria*), there is a robust molecular marker discriminating sexual and apomictic plants. The genes involved in *C. ciliaris* have been examined and compared with other species (Roche et al., 1999; Akiyama et al., 2005; Yadav et al., 2012). Similar to the widely grown *Urochloa* (*Brachiaria*) tropical forage grasses (Tomaszewska et al., 2021a,b), apomicts at all ploidies produce seeds, as there is neither meiosis nor necessity for restitution of diploid pairing behavior (Sepsi et al., 2017). Although there is relatively weak evidence that polyploid plants as a group outperform diploids either in wild or agricultural settings (particularly where selection is strong enough to remove mildly deleterious mutations), there are many prospects to exploit polyploidy systematically using additional gene alleles and increased genetic variability, which enhance adaptability (Brits et al., 2003; Bhat et al., 2005; Kharrat-Souissi et al., 2014).

*Cenchrus ciliaris* has a genome size of approximately 3,000 Mbp in the tetraploid species, 50% larger than the 1,971 Mbp reported for the genome of *C. purpureus* (Elephant or Napier grass; 2*n* = 4*x* = 28) and the more distantly related but smaller 1,580 Mbp *C. americanus* (2*n* = 2*x* = 14; syn. *Pennisetum americanum*) genome (Yan et al., 2021). A major proportion of plant genomes consist of repeated sequences such as ribosomal DNAs, tandem repeats, satellites, and transposable elements, which play an important role in the shaping of genome organization (rDNA in *Cenchrus*: Kharrat-Souissi et al., 2012; other species: Heslop-Harrison and Schwarzacher, 2011; Macas et al., 2011; Biscotti et al., 2015). Yan et al. (2021) reported the composition of the *C. purpureus* genome as comprising 66.32% repetitive sequences, similar to the proportion of repeats in *C. americanus* (77.2%). Ty3-*gypsy* and Ty1-*copia* are two superfamilies of LTR-retroelements widely distributed at the centromeric region of chromosomes in many members of Poaceae and terminal heterochromatic chromosomal regions in *Allium cepa*, respectively (Kumar et al., 1997).

Members of the major groups of repetitive elements, tandemly repeated satellites, and transposable elements (Class I retrotransposons and Class II DNA transposons) differ in their abundance and genomic distribution in the genome. Tandemly repeated motifs may be located in subterminal, intercalary, or centromeric regions of chromosomes, or, apart from the 5S and 45S rDNA arrays, not be abundant. In contrast, all plants have relatively abundant retrotransposons, often with a dispersed distribution (Heslop-Harrison et al., 1997), and often dispersed throughout the euchromatin but absent from specialized regions such as heterochromatin, centromeres, telomeres, and nuclear organization regions (NOR). Retrotransposon families may also be restricted to characteristic genomic locations. In *Arabidopsis thaliana*, many Ty3-*gypsy* retroelements are present in the pericentromeric heterochromatin region (Brandes et al., 1997b). Centromeric retroelements in maize (CRM) are a lineage of Ty3-*gypsy* retroelements (Jin et al., 2004), and the diploid oat *Avena longiglumis* (2*n* = 2*x* = 14; 3,850 Gb genome size) has specific LTR retrotransposons located at the centromeres (Liu et al., 2019, 2022); in the rest of the cases, they are present with other retroelement families dispersed along all chromosomes. Some of these repetitive elements may have a role in centromere functionality, although they may also accumulate in regions of the genome where there are few genes.

The activity of retroelements is modulated by epigenetic mechanisms through DNA methylation (Slotkin and Martienssen, 2007; Le et al., 2015), wherein the methyl group is covalently added to the fifth carbon of cytosine residues to form 5-methylcytosine (5-mC) (Moore et al., 2013). The expression profile of retroelements can be correlated with the differential methylation pattern (Castilho et al., 1999). DNA methylation is mainly distributed in heterochromatic regions in the genome that are rich in repetitive sequences consisting of retroelements (Vining et al., 2012). Comparison of methylomes between the tissues or of different stages can be studied to determine genes regulated through methylation and the pathways involved in response to stress and developmental conditions (Li et al., 2020). Whole-genome methylation analysis can reveal the distribution pattern of methylation in the genome and the associated genes or regions. In *A. thaliana*, genome-wide methylation analysis found that one-third of genes are methylated within their transcribed regions, while in rice, approximately 16% of genes are enriched with methylation (Zilberman et al., 2007; He et al., 2010; Vining et al., 2012). Genome methylation analysis in *Populus trichocarpa* also indicated that Ty3-*gypsy* retroelements, which are abundant in centromeric, pericentromeric, and heterochromatic regions in plants, are enriched with DNA methylation (Douglas and Di Fazio, 2010; Vining et al., 2012). Therefore, the methylation status of retroelements in the genome can be assessed by combining the physical mapping of retroelements through fluorescent *in situ* hybridization (FISH) with an assessment of 5-methylcytosine through immunostaining, which enables us to understand the genome-wide distribution pattern of retroelements and their association with the methylation.

In this study, we aimed to characterize the nature of repetitive DNA in *Cenchrus ciliaris*, investigate the genomic organization, including tandemly repeated satellite DNA and LTR retroelements, and examine genome-wide methylation patterns in *C. ciliaris*, aiming to characterize features related to its contribution to the genome, evolution, and possible variation in the genomes.

## Materials and methods

### Fixation of plant material

Seeds of *Cenchrus ciliaris* (CcA7-5) were germinated in Petri dishes at 25°C for 3 days, and root tips were collected. Seedling root tips were pretreated in alpha-bromonaphthalene for 2 h to accumulate metaphases and then fixed in ethanol:glacial acetic acid (3:1) solution and stored at 4°C (Schwarzacher and Heslop-Harrison, 2000).

### Preparation of chromosome spreads

Root tips were washed two times in enzyme buffer (10 mM citric acid/sodium citrate) for 15 min and then digested with enzyme solution (20 U/ml cellulase, 10 U/ml Onozuka RS cellulase, and 20 U/ml pectinase in enzyme buffer) for 45 min at 37°C. After digestion, root tips were again washed in enzyme buffer. The meristematic tissues were dissected and squashed in 60% acetic acid under the coverslip by using light thumb pressure. Slides were frozen on dry ice for 5–10 min and then coverslips were removed with a razor blade. Slides were air dried and used for FISH.

### Isolation of genomic DNA and designing of probes for repetitive sequences

Genomic DNA was isolated from the apomictic genotype *C. ciliaris* (CcA7-5) using the Qiagen DNeasy Plant mini kit. Primers were designed from conserved regions of the reverse transcriptase domain of LTR retroelements (Ty1-*copia* and Ty3-*gypsy*) and 45S ribosomal DNA genes using the BLAST search to identify the conserved region (Sayers et al., 2019; Table 1). Whole-genome sequencing data from a Chinese variety of *C. ciliaris* (deposited in SRA Sequence Read Archive under accession SRR8666664, Illumina HiSeq 2500; Nevill et al., 2020) were downloaded and used to discover the most abundant repetitive DNA sequences. Similarity-based clustering, repeat identification, and classification were performed using RepeatExplorer2 (Novak et al., 2013). In detail, raw paired-end reads were preprocessed, including quality-filtering (quality score ≥10 over 95% of bases and no Ns allowed), trimming, excluding overlapping read pairs, and interlacing. A subset of reads was randomly sampled from a dataset as input for RepeatExplorer to identify repetitive sequences and tandem repeats (following Novak et al., 2013, including the use of the REXdb reference database of transposable element domains, Neumann et al., 2019). For selected repetitive elements, primers were designed (Table 1) using Primer3 (Rozen and Skaletsky, 1999). Conserved regions were amplified from genomic leaf DNA in a standard polymerase chain reaction (PCR) using specific primers synthesized commercially (Sigma). DNA probes for Ty3-*gypsy*, Ty1-*copia*, CL105, CL58, and 18S rDNA were obtained by PCR amplification from genomic DNA, and labeled with biotin-16-dUTP or digoxigenin-11-dUTP with the BioPrime CGH array labeling kit (Invitrogen), and then purified using BioPrime Purification Module (Invitrogen). Fluorochrome-labeled synthetic oligonucleotide probes for the clusters (CL), CenchCL58_869, CenchStCL1ConsS, CenchEnCL1ConsRC, and CenchCL22Copia were used as probes for FISH (Table 1).

**Table 1:** List of PCR primers and commercial oligonucleotide probes used in the study.

**Primer name**	**Primer sequence**	**Oligonucleotide length [bp]**	**Product length [bp]**	**Probe name**	**References**
CopiaQMDVKT _F CopiaYVDDML_R	CARATGGARGTNAARAC ARCATRTCRTCNACRTA	17	290	universal Ty1-copia	Flavell et al., 1992a,b; Hirochika and Hirochika, 1993
GypsyRT1RMCVDYR_F	MRNATGTGYGTNGAYTAYMG	20	410	universal Ty3-gypsy	Friesen et al., 2001
GypsyRT4YAKLSKC_R	RCAYTTNSWNARYTTNGCR	19			
18S rDNA_F	CGAACTGTGAAACTGCGAATGGC	23	900	18S rDNA	Chang et al., 2010
18S rDNA_R	TAGGAGCGACGGGCGGTGTG	20			
CC_C105F76	CGAGTAGCCCAAATCCAGGA	20	590	Gy105	This paper
CC_C105R665	TTGCTGAACCAACTCCCTGT	20			
CC_C105F76	CGAGTAGCCCAAATCCAGGA	20	919	Gy105	This paper
CC_C105R994	CAAGGAAGGGGAAACAACGG	20			
CenchCL58_361F	GGCTGGGGGCGATAAGTAG	19	350	CenchCL58	This paper
CenchCL58_710R	CAAGAAGGCGCAGATTGTGG	20			
CenchCL58_869	[Cy3]CTGGAGGTTGTAATTCCAAGGCCACGCATCTCAGAAGGGAGTCG	44	–	CenchCL58_869	This paper
CenchStCL1ConsS	[Cy3]CCATTTTCACAACTTCGGTACCCTGATGTAGTGCATTCATGCAC	44	–	CenchStCL1ConsS	This paper
CenchEnCL1ConsRC	[FAM]GCCTACCCCATTAGATCCCAAACGATGTTTGAGAGTGTTTAGGAG	45	–	CenchEnCL1ConsRC	This paper
CenchCL22Copia	[FAM]GGGGTTAGAAATAGGACCCAACAAGAAAAACAATATGATGTAACCGCGAGG	51	–	CenchCL22Copia	This paper

### Fluorescence *in situ* hybridization

Slides were refixed in ethanol:glacial acetic acid (3:1) for 30 min, dehydrated in 100% ethanol two times for 5 min, and then air dried. Slides were treated with RNase (100 μg/ml) in 2X SSC for 1 h at 37°C in a humid chamber and then washed two times in 2X SSC for 5 min at room temperature. Then, the slides were incubated in 0.01 M HCl for 2 min, treated with pepsin (5 μg/ml) in 0.01 M HCl for 10 min at 37°C in a humid chamber, rinsed in distilled water for 1 min, and washed in 2X SSC for 5 min. Slides were refixed in 4% formaldehyde at room temperature for 10 min, washed in 2X SSC for 2 min, and then in 2X SSC for 10 min. Slides were then dehydrated in an ethanol series (70, 85, and 100% ethanol, 2 min each).

The hybridization mixture consisted of probes (2 ng/μl each; Table 1), 50% formamide (v/v), 2X SSC, 10% dextran sulfate (v/v), 200 ng/μl salmon sperm DNA, 0.125 mM EDTA, and 0.125% SDS; a total volume of 40 μl was denatured at 83°C for 10 min and then cooled down on the ice for 10 min. The hybridization mixture was then added to the slides and placed in a hybridization oven at 73°C for 6 min to denature the probe and chromosomes and then cooled to 37°C and left at that temperature overnight for hybridization.

Posthybridization washes were performed to remove the unincorporated and weakly hybridized probes. Slides were washed two times in 2X SSC for 2 min at 42°C and then washed in 0.1X SSC at 42°C for 2 min and again for 10 min at the same temperature before a final wash in 2X SSC for 5 min at room temperature. To detect probes, slides were incubated in a detection buffer (4X SSC containing 0.2% Tween 20) for 5 min and blocked by incubation with 5% BSA (bovine serum albumin) in a detection buffer for 10 min at 37°C in a humid chamber. Hybridization sites were detected by incubation with streptavidin conjugated to Alexa 594 streptavidin antibody or FITC (fluorescein isothiocyanate) anti-digoxigenin antibody in the detection buffer with 5% BSA for 1 h at 37°C in a humid chamber. Slides were washed in detection buffer three times at 40°C for 10 min each. Chromosomes were then counterstained with DAPI (4',6-diamidino-2-phenylindole) in antifade solution (AF1, Citifluor) and photographed.

### Methylation analysis using anti-5-methylcytosine through immunostaining

Immunostaining was performed to visualize how whole-genome cytosine methylation is distributed in different chromosomal regions of *C. ciliaris* genome. Coverslips were removed from slides previously denatured, probed with the DNA, hybridized, and analyzed. After washing in detection buffer, the preparations were blocked by 1% BSA (w/v) in 1X PBS buffer/0.5% Tween 20 for 30 min at room temperature and incubated with primary anti-5-methylcytosine antibody (anti-5-mC; 1:500 in 1X PBS buffer/0.5% Tween 20) for 1 h at 37°C in a humid chamber. Slides were then washed in 1X PBS buffer/0.5% Tween 20 two times for 5 min each at room temperature and incubated with Alexa 488-conjugated goat anti-mouse secondary antibody (1:500) in 1X PBS buffer/0.5% Tween 20. Slides were then washed two times in 1X PBS buffer/0.5% Tween 20 for 5 min at room temperature and stained with DAPI in antifade solution (AF1, Citifluor) and metaphases previously analyzed with FISH were rephotographed after immunostaining.

### Photography and imaging

Slides were examined with a Nikon Eclipse 80i epifluorescence microscope equipped with a DS-QiMc monochromatic camera and NIS-Elements version 2.34 software (Nikon). Images were analyzed using the Adobe Photoshop software.

## Results

### Chromosomes of *Cenchrus ciliaris*

**Figure 1:**
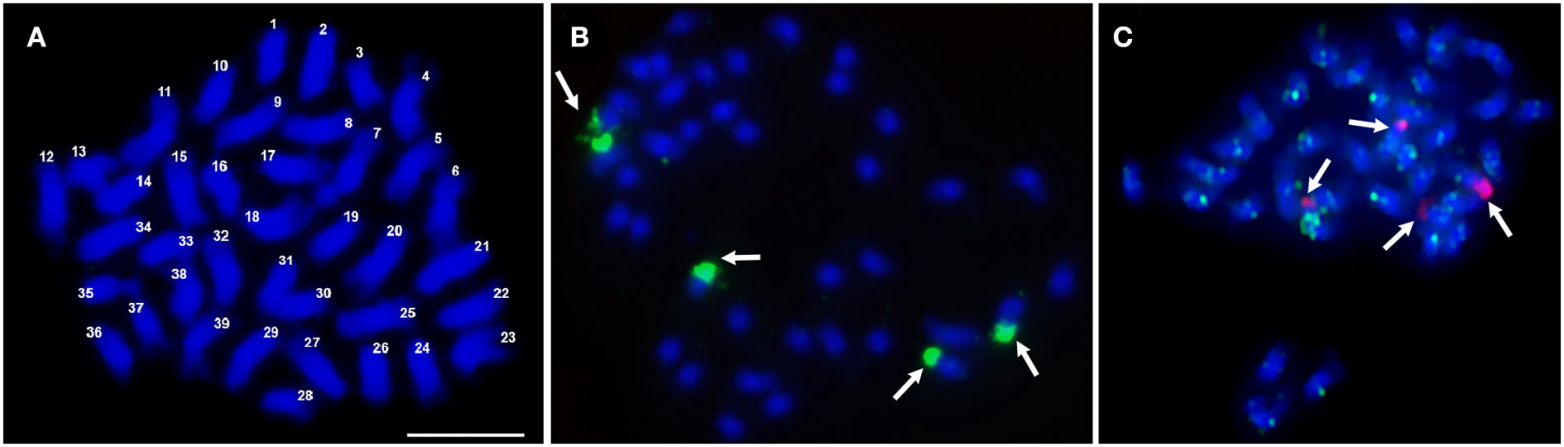
Number of chromosomes and rDNA sites in *Cenchrus ciliaris*. **(A)** Metaphase spread of *C. ciliaris* after DAPI staining (blue) showing a chromosome count of 2*n* = 4*x* + 3 = 39. **(B)** Fluorescent *in situ* hybridization with 45S rDNA probe fluorescing green (arrows). **(C)** 45S rDNA probe (red) showing four major hybridization sites and Gy105 probe (green) hybridizing to most parts of the chromosomes. Scale bar = 10 μm in **(A)**.

Most plants of the apomictic tetraploid *C. ciliaris* were 2*n* = 4*x* = 36, but one hyperploid 2*n* = 4*x* = 39 plant was found (Figure 1A); all chromosomes were submetacentric and of similar length. The 45S rDNA probe was located at four subterminal regions (Figures 1B,C arrows), also visible as the satellite at the nucleolar organizing region (NORs by DAPI staining). Similar results were also observed by Kharrat-Souissi et al. (2012).

**Figure 2:**
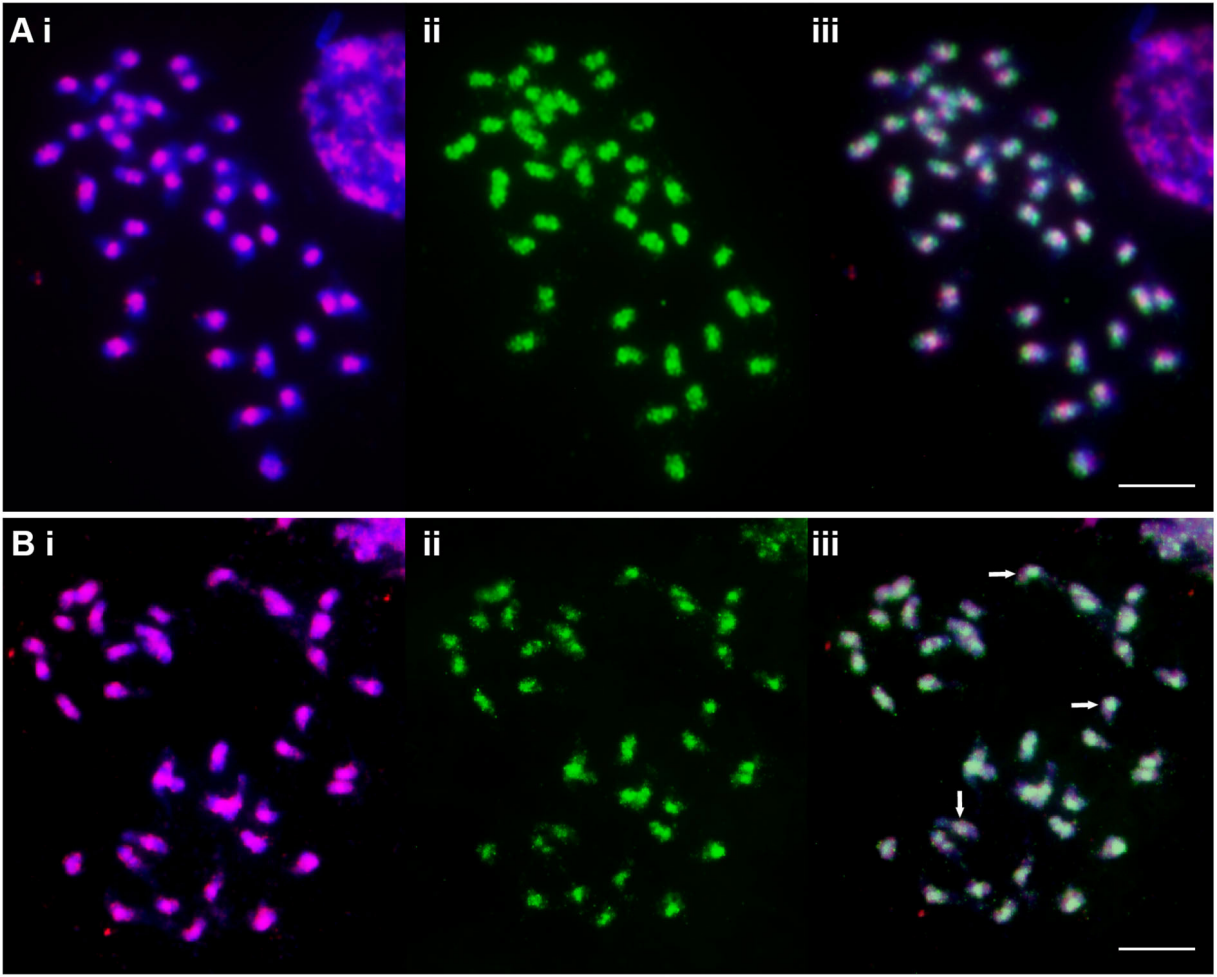
Metaphase chromosomes of *C. ciliaris* (2*n* = 4*x* + 3 = 39) after FISH (red fluorescing, **(i)** and immunostaining with anti-5-methylcytosine (green fluorescing, **(ii)** and as an overlap image **(iii)**. **(A)** The “universal” Ty1-*copia* probe shows concentrated signal around the centromeres of all chromosomes, while 5-mC signal is not only seen overlapping them but also further along the chromosome arms. 5-mC strength is variable between the chromosomes with most showing a small gap at the centromeres. **(B)** The “universal” Ty3-*gypsy* probe shows a dispersed signal over the chromosome arms, but is absent from the most distal parts. 5-mC signal covers most Ty3-gypsy signals but is weak or absent in some cases (arrows). Scale bar = 10 μm.

The degenerate PCR product used as a “universal” Ty1-*copia* (Table 1) probe labeled broad regions of chromosomes, with sites often overlapping or spanning centromeric DAPI-positive heterochromatin (Figure 2Ai). Along with the centromeric regions, the arms of most chromosomes were labeled with Ty3-*gypsy*, but most distal ends lacked signal (Figure 2Bi). Probe Gy105 (Table 1), amplified from a sequence including a *Cenchrus ciliaris* Ty3-gypsy retrotransposon (Genbank JN559989, extended from Genbank ED545466 identified in a BAC by Conner et al., 2008) (Figure 1C, green signal), labeled regions near centromeres on most but not all chromosomes.

Immunostaining with anti-5-methylcytosine antibody showed genome-wide methylation patterns in apomictic *C. ciliaris* along chromosomes, with variability observed in the patterns. About one-third of chromosome arms were more weakly labeled, particularly at the ends of the arms; most centromeric regions showed small gaps, and a few chromosomes were strongly labeled (Figures 2Aii, Bii).

Chromosomal locations of the anti-5-methylcytosine antibody were compared with retroelement distribution by sequential labeling of the same metaphase preparations. The anti-5-mC signals overlapped the centromeric Ty1-*copia* probe signals of most of the chromosomes (Figure 2Aiii). Immunostaining of chromosomal spread previously probed with Ty3-*gypsy* retrotransposons showed methylation signals of anti-5-mC overlapped with Ty3-*gypsy* retroelements on most of the pericentromeric and centromeric regions of chromosomes. However, some Ty3-*gypsy* signals on the arms of chromosomes did not overlap with anti-5-mC signals (Figure 2Biii; examples show arrows). Along chromosome arms, the anti-5-mC signal was generally more widespread than retroelement.

### Chromosomal organization of highly abundant repetitive DNA motifs

Graph-based clustering (RepeatExplorer) of the survey sequence of *Cenchrus ciliaris* (SRR8666664; Nevill et al., 2020) identified highly abundant repetitive DNA motifs in the genome (Supplementary Figure 1A). A total of 332,421 raw reads showed that approximately 50% of reads clustered into 13,278 superclusters and 165,079 singlets (Supplementary Figure 1A; full analysis including summary tables are available at http://dx.doi.org/10.25392/leicester.data.19798966). For *in situ* hybridization, three clusters with a circular graphical representation (CL22, CL58, and CL105) and one cluster (CL1, with a star-like graph pattern characteristic of tandem repeats) were used (Supplementary Figures 1B,C; Supplementary Table 1). Those with rDNA or organellar DNA similarities were excluded from further analysis. The extracted clusters were checked by BLAST for repeat identification (Sayers et al., 2019).

CL1 (Supplementary Table 1; Supplementary Figure 1B) accounted for 2.5% of the *C. ciliaris* genome and is a putative low-confidence satellite of 140 bp monomer length (Supplementary Figure 1C), part of which is homologous to a similarly sized satellite reported in *Setaria viridis* (Thielen et al., 2020). From two different parts of this sequence, two oligonucleotide probes (44 and 45 bp long) were designed, namely, CenchStCL1ConsS and CenchEnCL1ConsRC (Table 1). CL58 (Supplementary Table 1; Supplementary Figures 1B,C) is identified as a satellite with a repeat unit of 1,326 bp that represents a much smaller proportion, 0.061% of the genome, and PCR primers and a 44 bp probe were designed for this sequence (Table 1). CL22 (Supplementary Table 1; Supplementary Figures 1B,C) is identified as a 4,807 bp Ty1-*copia* retrotransposon, and the oligonucleotide probe CenchCL22Copia was designed to the RNaseH domain (RC) from this sequence (Table 1). The probes were localized on metaphase chromosomes of *C. ciliaris* using fluorescence *in situ* hybridization.

**Figure 3:**
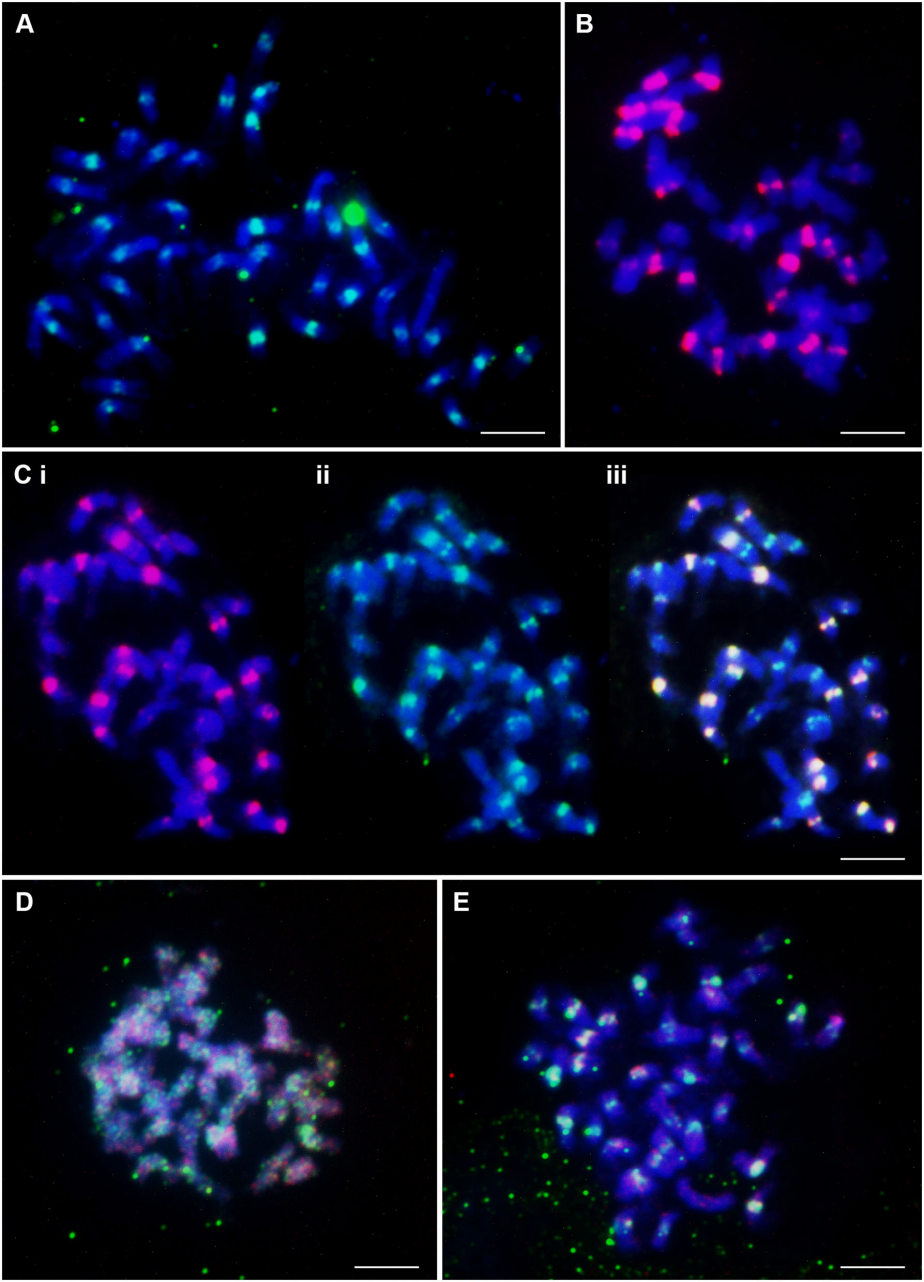
Distribution of highly abundant repetitive DNA motifs on chromosomes of *C. ciliaris* (2*n* = 4*x* + 3 = 39). **(A)** Tandem repeat CenchEnCL1ConsRC probe labeled with FAM (green) showed strong signals in pericentromeric regions of about half of the chromosomes. The remaining chromosomes showed weak signals. **(B)** Twenty very strong and four weaker red signals of Tandem Repeat CenchStCL1ConsS probe labeled commercially with Cy3 (red). **(C)** Three pictures of the same metaphase showing colocalization of CL1 and CL22 signals: **(i)** CenchStCL1ConsS (labeled with Cy3; red) showing strong signal on about half of the chromosomes, **(ii)** CenchCL22Copia (labeled with FAM; green) with strong signals on about half the chromosomes also showing strong CL1 signal, and weaker CL22 signal on the remaining chromosomes, **(iii)** merged. **(D)** Metaphase chromosomes showing dispersed signals of amplified CenchCL58 probe (detected with FITC; green) and synthetic labeled CenchCL58_869 probe (Cy3, red). **(E)** Dispersed signals of CenchCL58_869 probe (Cy3, red) along chromosomes. Some of the stronger signals in the image colocalize with CenchEnCL1ConsRC (FAM; green). Scale bar = 10 μm.

The CenchEnCL1ConsRC probe gave strong signals mostly in the pericentromeric regions of approximately 20 chromosomes from each metaphase, and the remaining chromosomes showed very weak signals (Figure 3A). In pericentromeric regions of chromosomes, 20 very strong signals of CenchStCL1ConsS probe were also detected, and some minor signals, mostly intercalary, were visible (Figure 3B). CenchStCL1ConsS colocalized with CenchCL22Copia signals (Figure 3C). Both probes are good chromosome markers, distinguishing about half of the chromosomes. The amplified CenchCL58 probe and commercially synthesized CenchCL58_869 showed dispersed signals along all chromosomes from metaphase (Figure 3D). Stronger signals of the CenchCL58_869 probe colocalized with CenchEnCL1ConsRC (Figure 3E), with 19 weaker and 20 stronger centromeric sites.

## Discussion

### Aneuploidy and polyploidy in *Cenchrus ciliaris*—consequences for forage grasses

*Cenchrus ciliaris* is an allotetraploid with chromosome number 2*n* = 4*x* = 36 (Akiyama et al., 2011) and a primary base number of *x* = 9. In this study, we reported a new chromosomal count of *C. ciliaris* with 2*n* = 4*x* + 3 = 39. Aneuploidy in *C. ciliaris* has been widely reported (Visser et al., 1998), with Burson et al. (2012) detecting 99 aneuploids from 568 accessions, with different chromosome numbers from 2*n* = 18 to 2*n* = 56 (Vij and Chaudhary, 1981). Hyperploidy or monosomic addition lines are well-known in tetraploids and hexaploids in many Poaceae members, including *C. ciliaris* (e.g., 2*n* = 6*x* + 1 = 55). Meiotic irregularities have been widely reported in *C. ciliaris*, presumably because of the close relationships of the ancestral genomes in the polyploids (Visser et al., 1998; Burson et al., 2012). Where seed production is continued through apomixis, the predominant commercial reproduction pathway in *C. ciliaris*, there is low selective pressure for euploids. Polyploidization has significant conservational and environmental consequences where one species can become invasive due to the adaptation of a species in a new locality and can also change reproductive biology (Thompson and Pellmyr, 1992; John et al., 2004). Thus, characterization of the genome constitution of apomictic *C. ciliaris* is important both genetically and environmentally.

Aneuploid and polyploid cytotypes are widely distributed within tropical forage grasses, including species of the genera *Cenchrus, Urochloa, Panicum, Pennisetum*, and *Paspalum* (Carloni-Jarrys et al., 2018; Worthington et al., 2019; Tomaszewska et al., 2021a,b), often arising from spontaneous chromosome doubling following nondisjunction after meiotic or mitotic divisions (Gallo et al., 2007; Burson et al., 2012; Worthington et al., 2019), and fertilization with unreduced (2*n*) gametes. Where seed is produced through apomixis, many ploidies and aneuploid lines can be maintained and remain unchanged (also maintaining any heterozygosis or heterosis in the maternal genotype; Lippman and Zamir, 2007), enabling propagation of heterozygous genotypes, which is valuable for grass breeding (Ozias-Akins and van Dijk, 2007). Unlike many tropical forage grasses, including the segmental allopolyploids (Figures 1, 2); Jessup et al., 2003; Worthington et al., 2019; Tomaszewska et al., 2021b), it has proved difficult to develop agronomically acceptable genotypes of cereal grain-crops (including *Triticum, Hordeum*, and *Avena* genera). Identification of loci that first control heterotic phenotypes and second parthenogenesis or apomixis in tropical forage grasses is important for forage grass improvement, and further work may lead to developing suitable systems to exploit apomixis in grain crops, regarded as a significant challenge for wheat and rice (Dresselhaus et al., 2001; Conner and Ozias-Akins, 2017; Rathore et al., 2020).

### Chromosomal localization of highly abundant repetitive DNA motifs

Various approaches have been used to identify and measure the abundance and genome organization of repetitive DNA in the plant genome. Assemblies from short reads will never provide the amount present because of the collapse of similar repeats during assembly (e.g., Yan et al., 2021). Graph-based clustering of unassembled raw reads, using RepeatExplorer (Novak et al., 2013), is proving to be a valuable reference-free approach to characterize all repeat families in a genome and identify those with genome specificity. Analysis of the frequency of k-mers—oligonucleotide sequences k bases long, where k is typically 16–64 bp—has also proved to be a robust method to identify repetitive motifs (Liu et al., 2019; Tomaszewska et al., 2021b).

In this study, we used both specific probes developed using graph-based sequence clustering compared with “universal” conserved regions of Ty1-*copia* and Ty3-*gypsy* retroelements. Similar to *Urochloa* tropical forage grasses, where genomic DNA and retroelements used as probes showed no substantial differences between the genomes (Santos et al., 2015; Tomaszewska et al., 2021b), parallel universal retroelement repeats used in this study were relatively equally abundant over the whole *Cenchrus ciliaris* genome (see Supplementary Figure 1). The Gy105 probe (see Figure 1D) labeled some chromosomes as more weaker. In contrast, CL1 (a putative satellite) and CL22 (an LTR-*copia* element) probes showed clear differential labeling of about half of the chromosomes (see Figure 3), and we suggest differences between the two ancestral genomes in the allopolyploid *C. ciliaris* having different retroelement (and/or satellite) sequence variants amplified before the genomes, which came together in the tetraploid. The “universal” retroelement probes amplified abundant sequences from both genomes and did not show any genome differentiation (see Figure 2).

### Methylation pattern and major repeat distribution

The genome distribution and organization of retroelements are important for understanding retroelement dynamics, movement or amplification, chromosomal structure, and evolutionary processes. In this study, centromeric DAPI-staining bands (corresponding to constitutive heterochromatin; Siljak-Yakovlev et al., 2002) collocated with Ty1-*copia* probes, suggesting accumulation of these retroelements at heterochromatic regions. Both Ty1-*copia* and Ty3-*gypsy* tended to cluster at specific regions on most of the chromosomes (along with some intercalary and dispersed sites) contrasting with other species (Brandes et al., 1997a) while Ty1-*copia* was more uniformly distributed on the euchromatin regions in the genome. Whether the nonuniform distribution of retroelements is due to insertion-site preference in the genome (Belyayev et al., 2001), perhaps of particular element subfamilies, or purging from genome regions, will require further study.

A combination of *in situ* hybridization with immunostaining using anti-5-mC allows correlation of the distribution of DNA methylation and sequence location across the genome (Sepsi et al., 2014). 5-mC has been extensively studied for its role in the regulation of gene expression, genome imprinting, and suppression of transposable elements (Zhang et al., 2010). In this study, anti-5-methylcytosine immunofluorescence overlapped with most of the Ty1-*copia* elements and Ty3-*gypsy*, showing their location predominantly in the methylated genomic regions associated with lower transcriptional activity. With the majority of 5-mC signals associated with retrotransposon-rich regions, there was no further differentiation of groups of chromosomes, and, in particular, no indication of differences between ancestral genomes in the polyploid. In contrast, differential methylation of parental genomes has been observed in intraspecific *A. thaliana* hybrids and implicated in heterosis as a key epigenetic factor (Lauss et al., 2018). However, in rice tetraploid hybrids, Li et al. (2019) reported overall methylome stability with regional variation of cytosine methylation states, in part associated with gene expression changes. Methylation repatterning, often stimulated by hybridization and/or polyploidization, can potentially lead to increased genetic variation through facilitating somatic recombination, which could be adaptive in apomictic lineages (Verhoeven et al., 2010). Changes in methylation patterns were observed both in the newly formed and in the established apomicts of *Taraxacum*, suggesting that considerable methylation variation can increase over short evolutionary time scales.

## Data availability statement

The datasets presented in this study can be found in online repositories. The names of the repository/repositories and accession number(s) can be found at: Previously published sequence data is available under accession SRR8666664 (Nevill et al., 2020). Results of the RepeatExplorer2 analysis of these sequence reads are archived on Figshare at http://dx.doi.org/10.25392/leicester.data.19798966.

## Author contributions

PR, VB, JH-H, and PT: funding acquisition. PR, TS, and PT: investigation and methodology. VB and JH-H: supervision. All authors: conceptualization, writing original draft review and editing, and have read and agreed to the published version of the manuscript.

## Funding

PR was supported under the Newton Bhabha Ph.D. placement program fellowship coordinated by the Department of Biotechnology, India, and the British Council, United Kingdom (BTIIN/UKJDBT-BC/2017-18). This study was supported under the RCUK-CIAT Newton-Caldas Initiative Exploiting biodiversity in *Brachiaria* and *Panicum* tropical forage grasses using genetics to improve livelihoods and sustainability, with funding from UK's Official Development Assistance Newton Fund, awarded by the UK Biotechnology and Biological Sciences Research Council (BB/R022828/1). PT has received support from the European Union's Horizon 2020 research and innovation program under the Marie Sklodowska-Curie grant agreement no. 844564 and no. 101006417 for the analysis of polyploid chromosomal evolution.

## Conflict of interest

The authors declare that the research was conducted in the absence of any commercial or financial relationships that could be construed as a potential conflict of interest.

## Publisher's note

All claims expressed in this article are solely those of the authors and do not necessarily represent those of their affiliated organizations, or those of the publisher, the editors and the reviewers. Any product that may be evaluated in this article, or claim that may be made by its manufacturer, is not guaranteed or endorsed by the publisher.

## Supplementary material

Supplementary Figure 1 Consensus sequences used to design primers and probes for fluorescence *in situ* hybridization. **(A)** RepeatExplorer graph showing number of raw sequencing reads forming abundant clusters; about 50% of the sequences form clusters, with <20 motifs representing one-third of the genome. **(B)** Graphical 2D projection of the structure of three graph-based clusters found in *Cenchrus ciliaris* genome using RepeatExplorer. Each node represents one of the sequence reads. The placement of the nodes reflects sequence similarity and overlaps. CL1 and CL58 are tandem repeats, while CL22 is an LTR-*copia* element. **(C)** Sequence LOGO representations showing lengths of repetitive motifs and conservation of sequences. CL1 is 140 bp tandem repeat; CL58 consensus is 1,326 bp and CL22 4,807 bp.

Supplementary Table 1 List of the most abundant clusters extracted from the Chinese variety of *Cenchrus ciliaris* SRR8666664 using RepeatExplorer.
