# Supplementary figures and images for: The repetitive DNA sequence landscape and DNA methylation in chromosomes of an apomictic tropical forage grass, Cenchrus ciliaris
Source: Front Plant Sci. 2022 Sep 15;13:952968. doi: 10.3389/fpls.2022.952968 (PMC9521199; doi:10.3389/fpls.2022.952968)

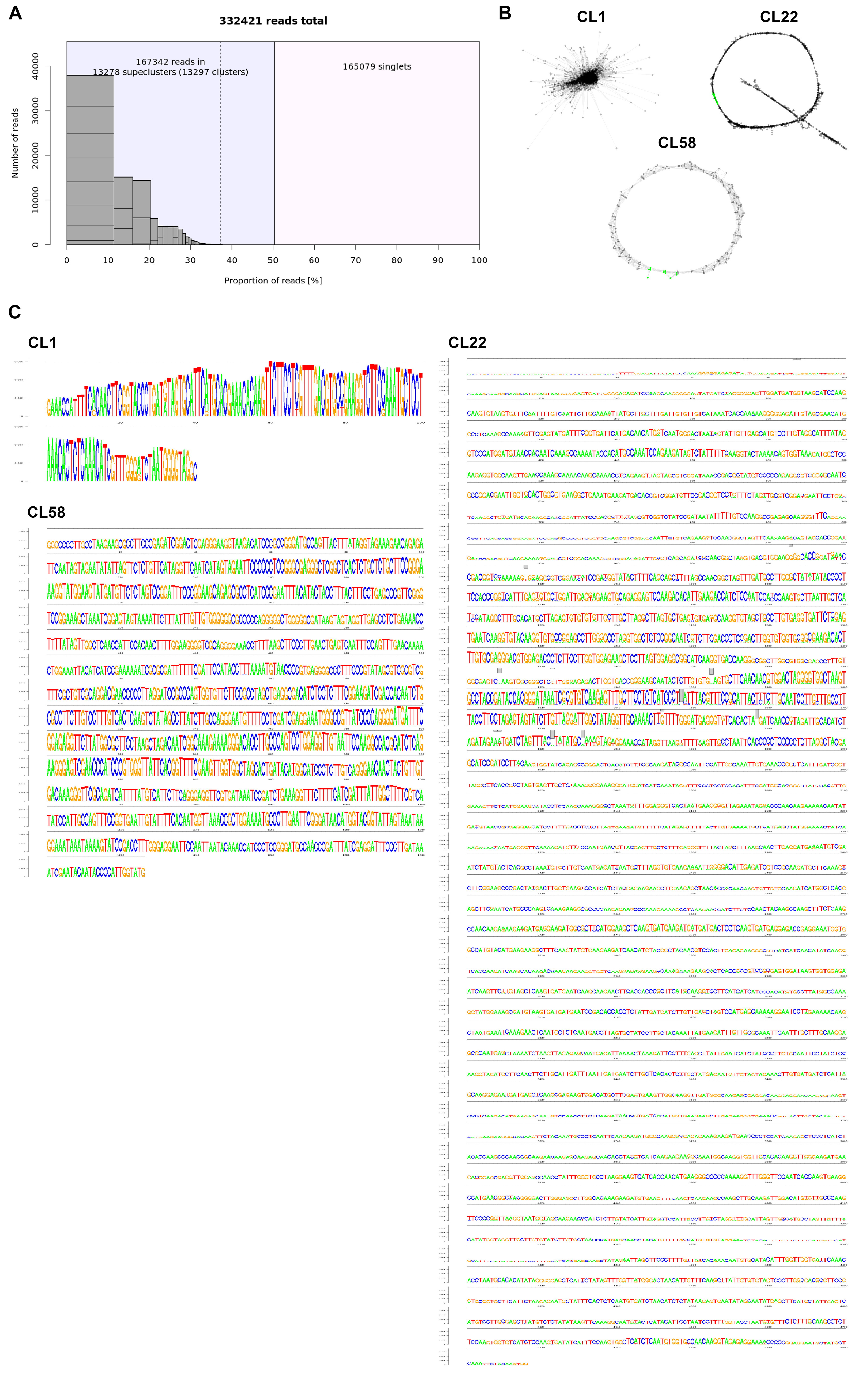

Supplement: Supplementary Figure 1 — Consensus sequences used to design primers and probes for fluorescence in situ hybridization. (A) RepeatExplorer graph showing number of raw sequencing reads forming abundant clusters; about 50% of the sequences form clusters, with <20 motifs representing one-third of the genome. (B) Graphical 2D projection of the structure of three graph-based clusters found in Cenchrus ciliaris genome using RepeatExplorer. Each node represents one of the sequence reads. The placement of the nodes reflects sequence similarity and overlaps. CL1 and CL58 are tandem repeats, while CL22 is an LTR-copia element. (C) Sequence LOGO representations showing lengths of repetitive motifs and conservation of sequences. CL1 is 140 bp tandem repeat; CL58 consensus is 1,326 bp and CL22 4,807 bp. [file Image_1.TIF]
