# Supplementary material for: The repetitive DNA sequence landscape and DNA methylation in chromosomes of an apomictic tropical forage grass, Cenchrus ciliaris
Source: Front Plant Sci. 2022 Sep 15;13:952968. doi: 10.3389/fpls.2022.952968 (PMC9521199; doi:10.3389/fpls.2022.952968)
Supplement: Supplementary Table 1 — List of the most abundant clusters extracted from the Chinese variety of Cenchrus ciliaris SRR8666664 using RepeatExplorer. [file Table_1.pdf]

Supplementary Data Table S1. List of the most abundant clusters extracted from the Chinese variety of *Cenchrus ciliaris* SRR8666664 using RepeatExplorer.

Name	Length [bp]	GC [%]	Sequence	Description
Cluster1	140	45.7	GAAACCATTTTCACAACTTCGGTACCCTGATGTAGTGCATTCATGCACGAAACAC<br>AAGTTCTTCCTCGTTTGACGTGCCGAAGGTCTTCCAAATGCTCCTAAACACTCTCA<br>AACATCGTTTGGGATCTAATGGGGTAGGC	Labelled oligonucleotide probes ordered are from 44bp (5 to 48; 46.7% GC) and RC of 45bp (96 to 140; 46.7% GC)
Cluster58	1326	47.4	GGGCCCCTTGCCTAAGAAGCGCCTTCCCGAGATCGGACTCGAGGGAAGGTAAGA<br>CATCCCGCCGGGATGCCAGTTACTTTATAGGTAGAAAGAACAGAGATTCAATAG<br>TAGAATATATTAGTTCTCTGTTCATAGGTTCAATCATAGTAGAATTCCCCCCTCCG<br>GGCGAGGGCTCGGCTCACTCTGCTCTGCTTCCGGGAAAGGTATGGAAGTATGATG<br>TTCTCTAGTCCGGATTTCCCGGAAGCAGAGCGCCTCATCCCGAATTTACATACTA<br>CCTTTACTTTCCTGAGCCCGTTCGGGTCCGGAAAGCTAAATCGGAGTAGTAAAAT<br>TCTTTATTTGTTGTGGGGGGCGCCCCCAGGGGGCTGGGGGCGATAAGTAGTAGGT<br>TGAGCCTCTGAAAACCTTTTATAGTTGGCTCAACCATTCCACAACTTTTGGAAGG<br>GGTCGCAGGGGAAACCTTTTAAGCTTCCCTTGAACTGAGTCAATTTCCAGTTTGA<br>ACAAAACTGGAAATTACATCATCCGAAAAAATCGCGCGATTTTTTCGATTCCATA<br>CCTTTAAAATGTAACCCCGTGAGGGGCCCTTTCCCGTATAGCGTCGCGTCGTTTC<br>GCTGTCGCAGGACGAACCCCCTTAGGATCCGCCCAGTGGTGTTCTTCCGCCTAGC<br>TGAGGCGACATCTCCTCTTTCGGAAGATCGACCACAATCTGCGCCTTCTTGTCCTT<br>TGTCACTCAAGTCTATAGCCTTATCTTGCCAGGGAATGTTTTCCTCGATCGAGGA<br>AATGGGCCGTTATCCCCAGGGGATGATTTCGGAGAGGTTCTTATGGCGCTTCCTA<br>AGCTAGACAATCGGCAAAGAAAAGGGACACCTTGCCCAGTCCTGGAGGTTGTAA<br>TTCCAAGGCCACGCATCTCAGAAGGGAGTCGAACCCATCCCGTGGGTTATTCACG<br>GTTTTCGAAGTTGGTGGCTAGCACTGATACATGGCATCCCTCTTGTCAGGGAACA<br>ACTACTGTTGTGACAAAGGGTTCGCAGATCATTTTATGTCATTCTTCAGGGAGGT<br>TCGTGATAAATCCGATCTGAAAGGTTTCTTTTCATCGATTTATTGGCTTTTCGTCA<br>TATCCATTGCCAGTTTCCGGTGAATTGTATTTTCACAATGGTTAAACCGCTGGAA<br>AATGCCCTTGAATTCGGGATAACATGGTACGGTATTAGTAAATAAGGAAATAAA<br>TAAAAGTATCCGACCTTTGGGAGGAATTCCAATTAATACAAACCATCCCTCCGGG<br>ATGCCAACCCGATTTATCGAGGATTTCCCTTGATAAATCGAATACAATACCCCAT<br>TGGTATG	Labelled oligonucleotide probe ordered is from 44bp (867 to 910; 54.5% GC)
Cluster22	4807	45.6	ACTTGCTAGATTCCAATAATATGAGTACCCTTTGGACACTTTTTGGAGATTTTATG<br>CCAAAGGGGGAGAGATAGTGGAGAGATCGTGGAGATTGGAGTCAAAGCAAGGC<br>AAGCATGGAGTAAGGGGGAGTGATCGGGGAGAGATCCAAGCAAGGGGGAGTAT<br>GATCTAGGGGGAGTTGGATGATGGTAAGCATCCAAGCAAGTGTAAGTGTTTCAA<br>TTTTGTCAATTCTTGCAAAATTATGCTTGCTTTGATTGTGTTGTCATAAATCACCA<br>AAAAGGGGGAGATTGTAGCGAACATGGCCTCAAAGCCAAAAGTTCGAGTATGAT<br>TTCGGTGATTCATGACAACATGGTCAATGGGACTAATAGTATTGTTGAGCATGTC<br>CTTGTAGGCATTTATAGGTCCCATGGATGTAACCACAATCAAAGCCAAAATACCA<br>CATGCCAAATCCAGAAGATAGTCTATTTTTCAAGGTACTAAAACAGTGGTAAAG<br>ATGGCTCCAAGAGGTGGCAAGTTGAACCAAAGCAAAACAAGCAAAACCTCAGA<br>AGTTAGTAGCGTCGGATAAACCGACGCTATGTCCCCCAGAGGCGTCGGAGCAAT<br>CGCCGGACGAATTGGTGCACTGGCGTGAAGGCTGAAATGAAGATGACACCGTCG<br>GATGTTCCGACGGTCCTGTTTCTAGTTGCGTCGGACGAATTCCTGATTCAAGGCT<br>GTGATGCAGAAGGCATCGGATTATCCGACGCTTGTAGCGTCGGTCTATCCGATAA<br>TATTTTTGTCCAAGGCCGAGAGCAAGGGTTTCAGGAACCCTTCAGCACCGGAAG<br>ATCCGACGCCCCGTCGGTGCAAGCGTCGGAGCAATTCTGTCAGAAGGTCCAACG<br>GCTAGTTCAAAAAGACAGTAGCACCGGATGAACCGACGGTGAAGAAAAGGCAG<br>CGTCGGACAAAGCGTCGGACAGATTGCGTCAGCAGATCGCAACGGCTAGGTGAC<br>GTGGAAGGGGCACCGGATAAACCGACGGTCCAAAAAGGGAGGCGTCGGAATAT<br>CCGATGGTATACTTTTCAGCAGCTTTTAGCCAACGGCTAGTTTGATGCCTTGGGCT<br>ATATATACCCCTTCCACCCGGTCATTTGAGTGTGCTGGATTGAGGAGAAGTGCAG<br>AGGAGTCCAAGACACATTGAAGACCATCTCCAATCCATCCAAGTGCTTAATTGCT<br>CATTCATAGGCTTTGCACATGCTTAGAGTGTGTGTGTTGCTTGCTTAGGCTTAGTG<br>CTGAGTGTGAGGCAAGGTGTAGCTGCCTTGTGAGGTGATTCTCGAGTGAATCAAG<br>GTGTACAAGGTGTGCCGGAGCCTTGGGGCCTAGGTGGCTCTCCGGCAATCGTCTT<br>CGACCCTCCGACTTGGTGTGGTGCGGCGAAGACACTTTGTGCGAGGGACGTGGA<br>GACCCTCTTCCTTGGTGGAGAAGCTCCTTAGTGGAGGCGGCATCAAGGTGACCAA<br>GGGCGGCTTGGCGTGGCGAGCCTTTGTGGCGAGTCAAGTGGCGGGCTCGTTGGG	Labelled oligonucleotide probe designed to RNaseH domain (RC) is from 51bp (2156 to 2206; 43.1% GC)

			AGAGACTTGGTGACCGGGAAGCAATACTCTTGTGTGAGTGCTTCAACAACGTGG<br>ACTAGGGGTGGCTAAGTGCCTACCGATACCACGGGATAAATCGCGTGTCAAGAG<br>TTTGCTTCCTCTCATCCCTATTTACGTTTCCGCATTACTTTCTTGCAATCCTTGTTT<br>GCCTTTACCTTCCTAGAGTAGTATCTTGTTAGGATTGGCTATAGGTTGCAAAACTT<br>GTTTTGGGATGAGGGTGTCACACTAGATCAACCGTAGATTGCACATCTAGATAGA<br>AATGATCTAGTTTACTATATGCAAAGTAGAGGAAACCATAGGTTAAGTTTTTAAG<br>TTGCCTAATTCACCCCCTCCCCCTCTTAGGCTACGAGCATCCGATCCTTACAAGTG<br>GTATCAGAGCCGGGACTCACATCTTTCGCAAGATACGCCAATTCCATTGGCAAAT<br>TGTGAAACCGGCTCATTTGATCGGTTAGGCTTCACCGCCTAGTGAGTTGCTCTAA<br>AGGGGAAAGGGATGGATCATCAAATAGGTTTCCCTCCTCCACATTTCGATGGCAC<br>GGGCTATCCACGTTGGAAAGTTCTCATGGAAGCTTACCTCCAAGCAAAGGGCCTA<br>AATGTTTGGAGGGTCACTAATGAAGGGGTTAGAAATAGGACCCAACAAGAAAAA<br>CAATATGATGTAACCGCGAGGAGCATCCTTTTGTCCTCTCTTAGTGAAGATGTTTT<br>TCATAGAGTTTTTACTTGTGAAAATGCTCATGAGCTATGGAAAACTATCAAAGAG<br>AATAATGAGGGTTCAAAAGATGTTTCCAATGAACGTTACGAGTTGCTCTTTGAGG<br>GTTTTACTAGCTTTAAGCAACTTGAGGATGAAAATGTCGAATCTATGTACTCACG<br>CCTAAATGTGCTTGTCAATGAGATTAATGCTTTAGGTGTGAAGAAAATTGGGGAC<br>ATTGAGATCGTCCGCAAGATGCTTCAAAGTCTTCGGAAGCCCGACTATGACTTGG<br>TGAAGTCCATCATCTACGAGAAGAAGCTTGAAGAGCTAACACCACAACAAGTGT<br>TGTGCAAGATCATGGCTCACGAGCTTCGAATCATGCCCAAGTCAAAGAAGGCGC<br>CCCAAGAGAAGCCCAAAGAAAAGCCTCAAGAACCATCTTCTCCAACTACAAGCC<br>AAGCTTTCTCAAGCCAACAAGAAAAGAAGATGAGGAAGATGGCGCTTCATGGAA<br>GCTCAAGTGATGAAGATGATGATGACTCCTCAAGTGATGAGGAGACCGAGGAAA<br>TGGTGGCCATGTACATGAAGAAGGCTTTCAAGTATGTGAAGAAGATCAACATGT<br>ACGGCTACAACGTCCACTTGAGAGAAGGGCGTCATCATCAACATATCAAGGTCA<br>CCAAGATCAAGCACAAAACCAAGAAGAAGGTGGTCAAGGAGAGGAAGCCAAAG<br>AAAGAAGCACTCACCGCCGTCCGCGAGTGGATAAGTGGTGGAGAATCAAGTTCT<br>TGTAGCTCAAGTGATGAATCAAGCAAGAACTTCACCACCCGCTTCATGCAAGGTC<br>CTTCATCATCATCCCACATGTGCCTTATGGCCAAAGGTATGGAAAGCGATGTAAG<br>TGATGATGAATCCGACACCACCTCTATTGATGATCTTGTTGAGCTAGTCCATGAG<br>CAAAAAGGAATCCTTGAAAAACAAGCTAATGAAATCAAAGAACTCAATGCTCTC<br>AATGACCTTAGTGCTATCCTTGCTACAAATTATGAAGATTTGTTGCGCAAATTCA<br>ATTTGCTTTGCAAGGAGCGCAATGAGCTAAAATCTAAGTTAGAGAGCAATGAGA<br>TTAAAACTAAAGATTCCTTTGAGCTTATTGAATCATCTATCCCTTGTGCAATTCCT<br>ATCTCCAAGGTAGATGCTTCAACTTCTTGCATTGATTTAATTGATGAATCTTGCTC<br>ACACTCTTGCTATGAGAATGTTGTAGTAGAAACTTGTGATGATCTCATTAGCAAG<br>GAGAATGATGAGCTCAAGCGAGAAGTGGACATGCTTCGAGTGAAGTTGGCAAGG<br>TTGATGGGCAAGAGCGAGGACAAGGAGGAACAAGAGGAAGTCCCTCAAGACAT<br>GAAGAGCAACGTCCAACCTTCTCAAGATAACCGTGATCACATGGTGAAGAAGCT<br>TGAGAAGGGTGAAACCGTGACTTGCTACAAGTGTCATGAAGAAGGGCACAAGTT<br>CTACAAATGCCCTCAATTCAAGAAGATGGGCAAGGGCGAGAGAAAGAAGATGA<br>ACCCCTCCATCAAGAGCTCCCTCATCTACACCAAGCCCAACCGCAAGAACAAGA<br>GCAAGAGCAACACCTATGTCATCAAGAAGAAGGCAAATGGCAAGGTGGTTGCAC<br>ACAAGGTTGGGAAGATGAAGACGGAGCGAGGTTGGAGCCAACCTATTTGGGTGC<br>CTAAGGAAGTCATCACCAACATGAAGGGCCCCCAAAAGGTTTGGGTTCCAATCA<br>CCAAGTGAAGGCCATGAACGGCTACGGGGACTTGGGAGGCTTGGCACAAAGAAG<br>ATGTGAAGTTTCAAGTCAAGAAGCCAAGCTTGCAAGATTGGACATGTGTTGCCCA<br>AGTTCCCCGGTTAAGGTAATGGTAGCAAGAACCCATCTCTTGTATCATTGTAGCT<br>CCATTGCCTTGTCTAGGTTTGCATTAGTTGTCATGCCTAGTTGTTTACATATGGTA<br>GGTTGCTTGTGTATCTTGTGCTAACCCATGAGCAACCTACATGTTTTGCCATGTGT<br>GTAGGAAATCTACACTTTGTTCTTTCATGGTGCATGCATTTCTTATGTTATCCTTT<br>GCATCATGAGCAAGCTATAGAATTAGCTTCCCTTTTGTTATCACAAACAATGTGC<br>ATACATTTGGTTGGTGATTCAAACACCTAATGCACACATATAGGGGGAGCTCATT<br>CTATAGTTTGGTTATGGGACTAACATTGTTTCAAGCTTATTGTGTGTAGTCCCTTG<br>GCGACGCGTTCCGGTGCGGTGCTTCATTCTAAGAGAATGCTATTTCACTCTCAAT<br>GTGATCTAACATCTCTATAAGAGTGAATATAGCAATATGAGCTTCATGCTATTGA<br>GTCATGTCCTTGCGAGCTTATGTCTCTATATAAGTTCAAAGGCAATGTACTCATA<br>CATTCCTAATCCTTTTGGTACCTAATGTGTTTCTCTTTGCAAGCCTCTTCCAAGTG<br>GTGTCATCTCCAAGTGATATCATTTCCAAGTGGCTCATCTCAATGTGGTGCCAAC<br>AAGGTAGAGAGGAAAACCCCCGGAGGAATGCTATGCTCAAATTCTACAAGTGG	
